# Assessment of a Targeted Trap-Neuter-Return Pilot Study in Auckland, New Zealand

**DOI:** 10.3390/ani8050073

**Published:** 2018-05-13

**Authors:** Sarah Zito, Glenn Aguilar, Shalsee Vigeant, Arnja Dale

**Affiliations:** 1 Animal Welfare Science and Education Department, Royal New Zealand Society for the Prevention of Animal Cruelty, 3047 Great North Road, New Lynn, Auckland 0640, New Zealand; arnja.dale@spca.nz; 2 Environmental and Animal Sciences, Unitec Institute of Technology, Carrington Road, Auckland 1026, New Zealand; gaguilar@unitec.ac.nz; 3 Royal New Zealand Society for the Prevention of Animal Cruelty, Auckland Shelter, 50 Westney Rd, Mangere, Auckland 2022, New Zealand; shalsee.vigeant@spca.org.nz

**Keywords:** trap-neuter-return (TNR), targeted trap-neuter-return, cat management, unwanted cats, shelter medicine, stray cats, semi-owned cats, animal welfare: shelter intake, shelter euthanasia

## Abstract

**Simple Summary:**

It is generally accepted that stray cats need to be managed to minimise the associated negative impacts and there is a need for effective and humane management tools. One such potential tool is trap-neuter-return (TNR), which anecdotally has been used in New Zealand to manage stray cats, but no concerted and targeted implementation of this technique has been reported, nor any formal assessments conducted. A targeted TNR (TTNR) programme for urban stray cats was implemented and assessed in one Auckland suburb. Assessment was based on the number of incoming felines; stray, unsocialised cats euthanased; unsocialised, unowned cats sterilised and returned (independently of the TTNR programme); and neonatal/underage euthanasias. Incoming stray feline, underage euthanasia, and unsocialised stray cat euthanasia numbers all reduced for the targeted suburb when these outcome measures were compared for the years before and after the programme. These outcome measures had a greater reduction in the targeted suburb compared to the other Auckland suburbs not targeted by the TTNR programme, although causation cannot be inferred, as a variety of reasons could have contributed to the changes. This pilot programme suggests that TTNR could be a valuable humane cat management tool in urban New Zealand, and further assessment is warranted.

**Abstract:**

There is a need for effective and humane management tools to manage urban stray cats and minimise negative impacts associated with stray cats. One such tool is targeted trap-neuter-return (TTNR), but no concerted implementation of this technique or formal assessments have been reported. To address this deficit, a TTNR programme was implemented and assessed in one Auckland suburb from May 2015 to June 2016; the programme sterilised and returned 348 cats (4.2 cats/1000 residents). Assessment was based on the number of incoming felines; stray, unsocialised cats euthanased; unsocialised, unowned cats sterilised and returned (independently of the TTNR programme); and neonatal/underage euthanasias. Incoming stray felines, underage euthanasias, and unsocialised stray cat euthanasias were all reduced for the targeted suburb when compared for the years before and after the programme (the percentage reduction in these parameters was −39, −17, −34, −7, and −47, respectively). These outcome measures had a greater reduction in the targeted suburb compared to the Auckland suburbs not targeted by the TTNR programme (*p* < 0.01), although causation cannot be inferred, as a variety of reasons could have contributed to the changes. This pilot programme suggests that TTNR could be a valuable, humane cat management tool in urban New Zealand, and further assessment is warranted.

## 1. Introduction

New Zealand is home to millions of cats; these cats are commonly classified as feral cats (cats that are not stray cats and have none of their needs provided by humans; generally do not live around centres of human habitation; and whose population size fluctuates largely independently of humans, is self-sustaining, and is not dependent on input from the companion cat population), stray cats (companion cats that have been lost, abandoned, or born stray; have many of their needs indirectly supplied by humans; live around centres of human habitation; and are likely to interbreed with the entire companion cat population), and companion cats (cats that live with humans as companions and are dependent on humans for their needs) [1,2]. The cats in these different populations differ considerably in terms of their lifestyle and interaction with humans, and management approaches need to reflect this. This paper focuses on cats in the stray category. Recently, the New Zealand National Cat Management Strategy Group (NCMSG) recommended that the cat categories be further clarified and refined to better reflect the cat populations that exist in New Zealand and in order to recognise the need for cat category-specific management. Most significantly, this involves refining the stray cat category to include managed and unmanaged stray cats [3]. Unmanaged stray cats do not have humans caring for them and any of their needs that are met by humans are indirectly supplied; conversely, managed stray cats are fed and cared for by people [4,5,6]. Managed stray cats have also been termed ‘semi-owned cats’ in the literature [4,6,7] and may also be called ‘colony cats’ if they live as a group of cats [8,9,10,11]. Although it is not possible to accurately quantify the stray cat population in New Zealand, estimates indicate that there are approximately 196,000 stray cats [12]. There are approximately 1,134,000 owned cats in New Zealand, making cats the country’s most popular companion animal [13].

Free-roaming cats, particularly stray and feral cats, are recognised as a significant issue in New Zealand [14], largely due to their impact, or potential impact, on native wildlife [12,15,16,17,18,19,20]. Stray cats can also be associated with many societal issues including ethical concerns about the euthanasia of thousands of healthy cats and kittens every year, and about the humaneness of cat control methods [21,22,23]; the moral stress and negative impact on the people involved in cat control [22,23,24,25]; financial costs to organisations that manage stray cats [3,26]; environmental and biodiversity costs [26,27,28]; potential for disease spread [3,26]; community nuisance [3,29]; and welfare concerns for the cats [30,31].

It is generally accepted that cats must be managed in order to mitigate the potential negative impacts of cats on communities, other species, and on the environment, but it is necessary to humanely manage cats in a way that also protects their welfare and, where possible, takes into account the interests of the humans involved in caring for the cats [3,6,32,33,34]. Traditionally used trap and remove, and trap and kill, programmes generally have not adequately addressed the issues associated with the negative impact of cats or the effect of cat control on cat welfare [3,35,36,37]. Although lethal control methods remove cats, computer models show that lethal control will fail to eliminate cat populations unless it is possible to consistently achieve high removal rates for long periods of time and control new cat immigration into the area [8,9,10,38,39,40]. In urban areas, this is unlikely to be feasible due to owned cats being affected, community resistance to lethal control methods, and such control methods being perceived as inhumane [26,41,42,43,44]. A recent study of public attitudes towards cat control in New Zealand indicated a preference for non-lethal control [44]. One such management tool that is increasingly being used internationally is trap-neuter-return (TNR) [37,45]; however, much controversy exists around the use of TNR [33,45]. Some studies have reported positive [5,11,46,47,48,49] and some negative [31,50] outcomes of TNR programmes, although the measures of success used to assess the outcomes differed between studies. None of these reports are from New Zealand, but anecdotally this management tool is being used in the country on an ad-hoc basis, but with no formal assessment reported. If this management tool is to be considered for more widespread use as a cat management tool in this country, it is important to assess its efficacy under New Zealand conditions. To the authors’ knowledge, this pilot study is the first effort to implement and assess a targeted TNR (TTNR) programme for urban stray cats in New Zealand. Although this pilot programme is short term in nature, and the assessment is based on shelter outcomes rather than population measures, the results will provide a valuable contribution to the discussion about cat management in New Zealand and internationally.

## 2. Materials and Methods

There are many animal shelters across New Zealand that accept, care for, and, where possible, rehome cats (and other animals). For some time, the shelter in Auckland that participated in this study had, where possible, and on an ad-hoc basis, been sterilising unsocialised stray cats that were brought to the shelter by members of the public but were unsuitable for rehoming, as an alternative to humanely killing these cats. This was only done if the cat was healthy and had been brought to the shelter by a member of the public who agreed to have the sterilised cat back and to continue to care for the cat. In addition to the ad-hoc sterilisation of unsocialised stray cats as previously described, a decision was made by the shelter management to implement and evaluate a targeted approach to sterilisation of stray cats (a targeted trap-neuter-return programme (TTNR)). This approach was taken to assess whether this might be an effective way to reduce the number of incoming stray cats and euthanasias in the shelter.

Ethics approval was not required, as this was a retrospective study that purely assessed the changes in shelter data associated with a TTNR programme being run as part of a normal shelter operation. Although the programme is described in detail in the methodology, the programme itself was being run not as a research project; the research solely assessed one programme within the overall shelter operation.

The suburb chosen for the TTNR pilot program was Manurewa; this suburb has 22,752 occupied dwellings [51] and was contributing the second highest number of incoming cats to the shelter. The targeted suburb was divided into 14 zones using street blocks, the postcode boundary, and natural boundaries such as main roads, motorways, and train tracks. Each zone was targeted by the programme for approximately one month, and the programme ran from May 2015 to June 2016 (with a month’s pause over the Christmas period due to lack of volunteer availability at this time).

A local veterinary clinic agreed to sterilise the trapped cats, and the clinic was paid a set amount for each surgery or euthanasia; the costs of any treatments were also agreed. This veterinary clinic performed all the sterilisation surgeries for the TTNR pilot programme.

Protocols were prepared by the shelter team for the programme; these included:

- Guidelines on which trapped animals could be sterilised, which animals should be released immediately (for example, owned cats and species other than cats), which animals could be treated if they were sick or injured, and which animals should be euthanased on humane grounds (if they were sick or injured and did not meet the criteria for treatment). Trapped kittens that were estimated to be 16 weeks of age or less were taken to the animal shelter and went through the normal procedure for rehoming if they were suitable.
- Tracking sheets to be used by volunteers and veterinary staff; these were completed for every cat in the programme. The tracking sheets included information about the trapped animal and where he/she was trapped, actions taken (i.e., release or transport to veterinary clinic), transport to veterinary clinic, admission to the veterinary clinic, physical examination findings, veterinary medical intervention (sterilisation and/or treatment, or euthanasia), discharge from the veterinary clinic, and return of the cat to the initial trap site.
- Protocols for treatment of programme cats at the collaborating veterinary clinic. These included guidelines on treatments that could be provided to programme cats with health problems (for example, abscesses or severe flea burdens), the need to use absorbable sutures and pre- and post-operative pain relief, the requirement that all sterilised cats to be released must be ear tipped, and the amount of time to hold cats in hospital prior to release (dependent on the cat’s situation, for example, castration of a healthy cat vs ovariohysterectomy of a pregnant cat).

Flyers explaining the programme were dropped in all of the mail boxes in each zone of the targeted suburb. This flyer advised residents of the dates when trapping would be performed in their area and invited those who had stray cats on their property to participate in the programme. To participate, the resident needed to be willing to have traps set on their property for the stray cats and then have the same cats returned to their property for ongoing care once the cats had been sterilised. If people had young kittens (estimated to be under 16 weeks of age) on their property they were advised that the shelter would take these animals and rehome them if possible. The flyers also advised cat owners that they should make sure that their cats had identification (collar and/or microchip) and included a paper collar that people could use to identify their cat. It was also suggested that cat owners keep their cats indoors during the period of cat trapping in their area.

Cat trapping was performed on set dates by volunteers who set traps with rain covers on the agreed properties in the evenings. These traps were then checked early in the mornings, and any cats that met the criteria of the programme were transported to the veterinary clinic for sterilisation. Any companion cats (cats that were collared, ear tipped, already sterilised, microchipped, had signs of previous medical attention, or were known to be owned from information gathered from the community) that were mistakenly trapped were released immediately. Any other species inadvertently trapped were also released if they were uninjured and healthy.

In order to evaluate the outcomes of the programme, data were extracted from the electronic shelter records for the financial years prior to, during, and after the programme (2014/2015, 2015/2016, and 2016/2017). For the purpose of this study, a juvenile cat was defined as one with a known or estimated age of six months or less and an adult cat was defined as one with a known or estimated age of greater than six months. The following measures were used to evaluate the TTNR project using data from the shelter records:

- The number of incoming adult and juvenile stray cats to the shelter from the targeted postcode, as an indication of the number of stray cats in that area.
- The number of neonatal/underage euthanasias performed at the shelter, as an indication of the number of kittens being born to stray cats in the community.
- The number of stray cats euthanased at the shelter because they were unsocialised and not suitable for rehoming, as an indication of the number of unsocialised stray cats.
- The number of unsocialised stray cats that were sterilised through the shelter after being brought in by a member of the public (a continuation of the previous shelter efforts to reduce healthy stray cat euthanasias, rather than as part of the TTNR programme). This is another indication of the number of unsocialised stray cats in the community.

These same measures were also compared between the targeted suburb and the other suburbs that were not targeted, as the participating shelter receives cats from the entire Auckland area.

The participating shelter also ran free sterilisation programmes for owned cats during the same time as the TTNR pilot; this is identified as a potential confounding factor and is elaborated on in the discussion.

### Statistical Analyses

The location of where the cats in the TTNR programme were trapped was recorded by suburb; this was geocoded using Google Fusion tables [52] to generate coordinates for each cat record. All of the cat records had suburb of origin information and, consequently, were able to be geocoded. The suburb of origin locations were then mapped with ArcMap10.5 [53,54,55]; as location records provided for the cats consisted only of suburbs and not exact geographical locations of the cats, the geocoding resulted in points located at the centroid of the polygon of the particular suburb. This is sufficient for comparing the spatial patterns over the entire study area using the cats per suburb as the major variable. Given the non-location specific nature of the data collected, this approach was also employed in using data of a similar nature [52], and the data sets for incoming cats, neonatal/underage euthanasias, unsocialised stay cats sterilised and returned, and unsocialised stay cats euthanased were joined with the locations. Each of the data sets consisted of juveniles, adults, and total cats for the 2014/2015, 2015/2016, and 2016/2017 financial year periods, except for neonatal/underage euthanasias, which only included juveniles. The percentage change from 2014/2015 to 2016/2017 for each of the outcome measures was also joined with the locations. The data for each of the outcome measures was then mapped on each location with column charts symbology representing the adult and juvenile cat numbers for each financial year period. This allowed the visualisation of trends and a comparison between adult and juvenile numbers throughout the study periods and also between locations.

A kernel density map was then created for all the data sets to show areas on the map of Auckland where greater numbers for each of the outcome measures were found. The kernel density tool of ArcMap fits a smooth surface over all the points using the values of the variable modelled [56]. The displayed options selected were the predicted density values, and for generating the surface, the planar method was used.

The Hotspot Analysis tool of ArcMap that calculates the Getis-Ord Gi* statistic was used to characterise the counts for each of the outcome measures in terms of the values of neighbouring data points [57]. The statistic identifies points with high values surrounded by points with similarly high values; these are designated as hot spots. Conversely, low values surrounded by low values are designated as cold spots. The statistical significance of each point in terms of its neighbourhood values results provides the basis for determining a level of confidence that a particular location is a hot spot, a cold spot, or that there is no significance in terms of this measure.

The percentage change in the number of incoming stray adult and juvenile felines, neonatal/underage euthanasias, unsocialised stray cats euthanased, and unsocialised stray cats sterilised and returned were each averaged for the non-targeted Auckland suburbs, as a group. The percentage change in the number of incoming stray adult and juvenile felines, neonatal/underage euthanasias, unsocialised stray cats euthanased, and unsocialised stray cats sterilised and returned were then all compared between the suburb targeted by the TTNR programme (Manurewa) and the average for these outcome measures for the non-targeted suburbs as a group. This comparison was performed with a Kruskal-Wallis analysis using the Real Statistics add-on for Excel [58].

## 3. Results

In total, 545 animals were trapped over the study period from May 2015 to June 2016; of these, 533 were cats, but 21 were companion cats; 91 were already ear tipped and/or sterilised and so were released immediately, 65 were kittens under 16 weeks of age that were taken to the shelter for rehoming, and 12 were animals of another species (hedgehogs and possums) that were unintentionally trapped and released immediately. That left 364 trapped cats that were suitable for inclusion in the programme and were taken to the veterinary clinic; 348 were sterilised and returned, seven were euthanased, and one was trapped again after her surgery due to a postoperative wound complication and was successfully treated and re-released. In addition, seven of the kittens taken to the shelter were sterilised and returned, as they were considered unsuitable for rehoming due to their unsocialised behaviour. No cats died peri-operatively.

The 2013 New Zealand census reported that 82,242 people usually live in the Manurewa Local Board Area in 22,752 occupied dwellings [51]. Based on these statistics, the pilot programme sterilised 348; this represents 4.2 cats/1000 residents. 

The decision to euthanase animals (sixteen at the shelter and seven at the veterinary clinic) was made for a variety of reasons in accordance with the programme guidelines. These reasons were that two cats were underage; two had ataxia, liquid diarrhea, and were underage; three had ringworm; three had ringworm and upper respiratory tract infection; two had severe oral ulcers and ringworm; two had chronic bronchitis/asthma and severe upper respiratory tract infection signs; one had self-trauma due to mites and allergies; one had advanced feline immunodeficiency virus; one had visible cancerous lesions; one had severe corneal ulcers; one had severe dehydration and emaciation; and one had severe oral ulceration.

### 3.1. Incoming Stray Felines

The year prior to the TTNR programme being implemented, 158 adult cats and 250 juveniles entered the shelter from the targeted suburb (Manurewa). This represents 5 incoming cats/1000 residents. The year of the programme, 122 adult cats and 274 juveniles entered the shelter from the targeted suburb (4.8 incoming cats/1000 residents). The year after the programme, this decreased to 96 adult cats and 207 juveniles (3.9 incoming cats/1000 residents). This represents an overall decrease of 39% for adult cats and 17% for juveniles between the year before and the year after the TTNR pilot programme. Please note that this change is likely to have been influenced by a number of different factors, which include but are not limited to the TTNR programme; this is elaborated on in the discussion.

**Figure 1:**
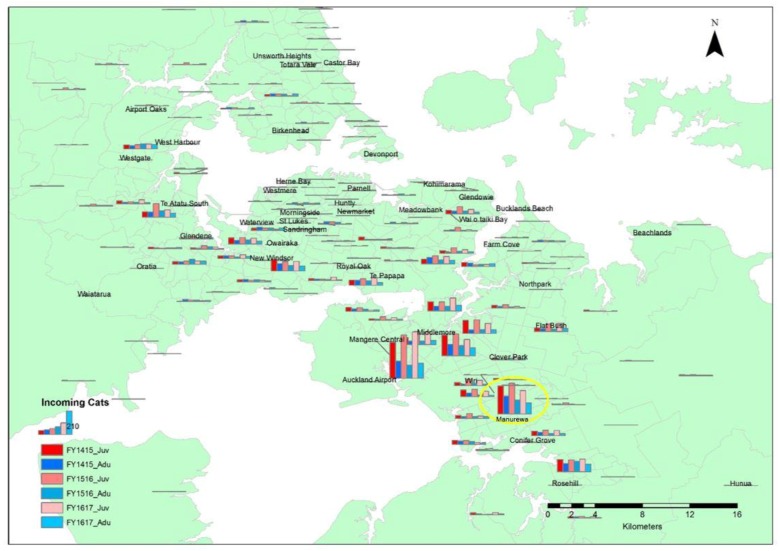
Map of Auckland showing the distribution of the origin of incoming juvenile and adult stray cats for the 2014/2015, 2015/2016, and 2016/2017 financial year periods across Auckland. (FY1415_J_2 and FY1415_A_1 = incoming juvenile and adult stray cats during the 2014/2015 financial year period, FY1516_J_2 and FY1516_A_1 = incoming juvenile and adult stray cats during the 2015/2016 financial year period, and FY1617_J_2 and FY1617_A_1 = incoming juvenile and adult stray cats during the 2016/2017 financial year period). The yellow circle highlights the data for Manurewa, the suburb targeted by the targeted trap-neuter-return (TTNR) pilot programme.

The column chart map of Auckland showing adults, juveniles, and total incoming cats for the 2014/2015, 2015/2016, and 2016/2017 financial year periods highlights much larger magnitudes in South Auckland, with the highest at Mangere Central. For Mangere Central, juveniles show an increasing trend, with numbers consistently more than double the adult numbers for the three periods (Figure 1). These data are shown in Figure 1, in which the column chart sizes indicate the quantities of the incoming cat data, with bigger graphs indicating greater quantities compared to the smaller graphs. This presentation immediately shows areas with higher magnitudes of incoming stray cat data per year recorded and also highlights the information for Manurewa.

### 3.2. Neonatal/Underage Euthanasias

The year prior to the TTNR programme being implemented, there were 68 neonatal/underage euthanasias from Manurewa. This represents 0.8 neonatal euthanasias/1000 residents. The year of the programme this decreased to 54 (0.7 neonatal euthanasias/1000 residents) and the year after 45 (0.6 neonatal euthanasias/1000 residents). This represents an overall decrease of 34% between the year before and the year after the TTNR pilot programme.

**Figure 2:**
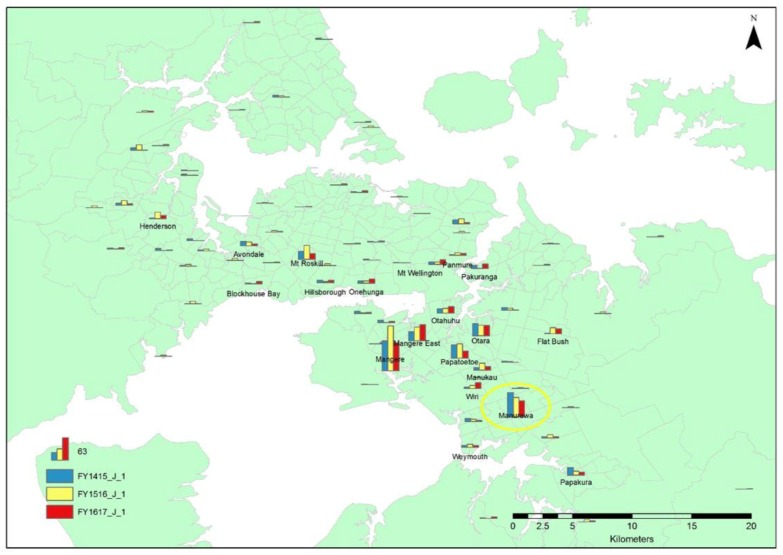
Map of Auckland showing the distribution of the origin of neonatal/underage euthanasias for the 2014/2015, 2015/2016, and 2016/2017 financial year periods. (FY1415_J_2 and FY1415_A_1 = neonatal/underage euthanasias during the 2014/2015 financial year period, FY1516_J_2 and FY1516_A_1 = neonatal/underage euthanasias during the 2015/2016 financial year period, and FY1617_J_2 and FY1617_A_1 = neonatal/underage euthanasias during the 2016/2017 financial year period). The yellow circle highlights the data for Manurewa, the suburb targeted by the TTNR pilot programme.

The column chart map of Auckland shows that the highest number of neonatal/underage euthanasias originated in the suburb of Mangere; the highest number was recorded in the 2015/2016 period, but the number then decreased in the 2016/2017 period. Manurewa was the area of origin for the second highest number of neonatal/underage euthanasias in 2014/2015, but then showed a steady decline in the following periods. The majority of the higher numbers of neonatal/underage euthanasias consistently originated in South Auckland for duration of the data (Figure 2). There were fluctuations in neonatal/underage euthanasias from many different suburbs over the assessed time period.

### 3.3. Unsocialised Stray Cats That Were Sterilised through the Shelter and Returned

**Figure 3:**
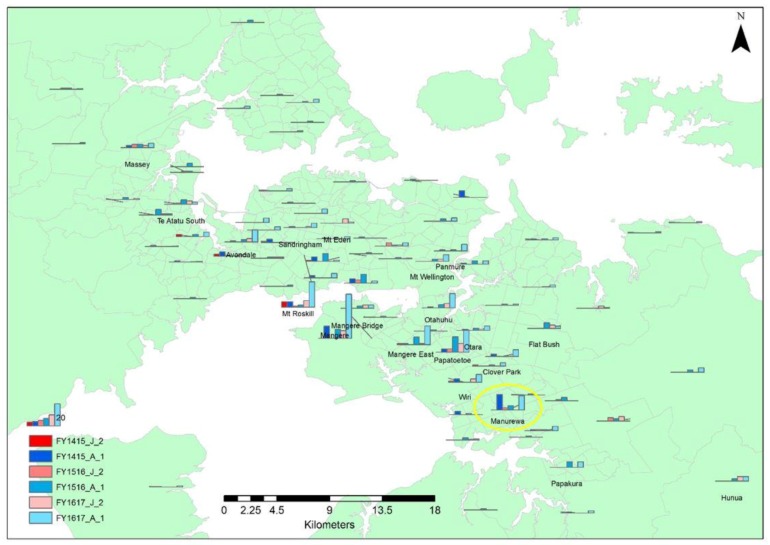
Map of Auckland showing the distribution of the origin of unsocialised adult and juvenile stray cats that were sterilised and returned through the shelter for the 2014/2015, 2015/2016, and 2016/2017 financial year periods. (FY1415_J_2 and FY1415_A_1 = juvenile and adult unsocialised stray cats respectively that were sterilised and returned during the 2014/2015 financial year period, FY1516_J_2 and FY1516_A_1 = juvenile and adult unsocialised stray cats, respectively, that were sterilised and returned during the 2015/2016 financial year period, and FY1617_J_2 and FY1617_A_1 = juvenile and adult unsocialised stray cats, respectively, that were sterilised and returned during the 2016/2017 financial year period). The yellow circle highlights the data for Manurewa, the suburb targeted by the TTNR pilot programme.

The year prior to the TTNR programme being implemented, 14 unsocialised adult stray cats from Manurewa were sterilised at the shelter and then returned (no juveniles) (0.2 unsocialised stray cats that were sterilised through the shelter and returned/1000 residents). In the year of the TTNR programme, four adult cats and two juveniles from Manurewa were sterilised and returned (0.1 unsocialised stray cats that were sterilised through the shelter and returned/1000 residents), in addition to those in the programme, as part of the ongoing efforts of the shelter to, where possible, identify, sterilise, and return appropriate cats that were brought directly into the shelter by members of the public and would have been euthanased otherwise. The year after the programme, there were 13 adult cats sterilised and returned (no juveniles) (0.2 unsocialised stray cats that were sterilised through the shelter and returned/1000 residents) (Figure 3).

The column chart map of Auckland illustrates that the 2016/2017 financial year period had a greater magnitude of adult stray cats sterilised and returned compared to other periods and also consistently more adults sterilised and returned compared to juveniles. The South Auckland area shows the highest number of stray cats sterilised and returned, but the number of adult stray cats sterilised and returned from Mt Roskill (which is situated in the Central Auckland area) shows a count that is comparable to many South Auckland locations (Figure 3).

### 3.4. Unsocialised Stray Cats Euthanased

The year prior to the TTNR programme being implemented, 46 adult cats and 9 juveniles from Manurewa were euthanased, because they were considered to be unsocialised strays and unsuitable for rehoming (0.7/1000 residents). The year of the programme, 25 adult and 7 juvenile unsocialised stray cats were euthanased at the shelter (separate from the TTNR programme) (0.4/1000 residents). The year after the programme, 16 adult and 13 juvenile unsocialised stray cats were euthanased (Figure 1) (0.4/1000 residents). This represents a decrease between the year before and the year after the TTNR pilot programme of 65% for adult cats, but an increase of 71% for juveniles from Manurewa.

**Figure 4:**
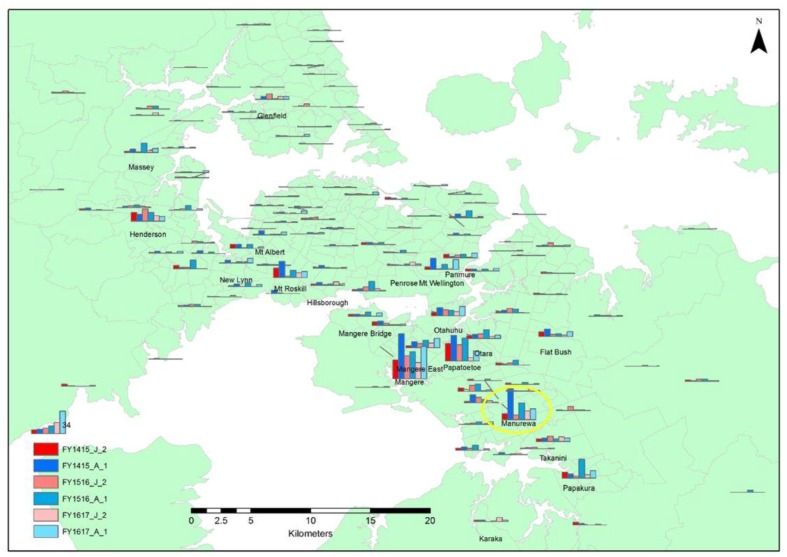
Map of Auckland showing the distribution of the origin of unsocialised adult and juvenile stray cats that were euthanased during the 2014/2015, 2015/2016, and 2016/2017 financial year periods. (FY1415_J_2 and FY1415_A_1 = juvenile and adult unsocialised stray cats, respectively, that were euthanased during the 2014/2015 financial year period, FY1516_J_2 and FY1516_A_1 = juvenile and adult unsocialised stray cats, respectively, that were euthanased during the 2015/2016 financial year period, and FY1617_J_2 and FY1617_A_1 = juvenile and adult unsocialised stray cats, respectively, that were euthanased during the 2016/2017 financial year period). The yellow circle highlights the data for Manurewa, the suburb targeted by the TTNR pilot programme.

The column chart map of Auckland illustrates that Mangere was consistently the suburb of origin for the highest number of both juvenile and adult unsocialised stray cats euthanased, followed by Papatoetoe and Manurewa. The number of unsocialised stray adults originating from Mangere that were euthanased increased in 2015/2016 and then decreased in 2016/2017, while the number of juveniles decreased and then increased over the same periods. Papatoetoe showed a marked decrease in both unsocialised stray adults and juveniles euthanased in 2016/2017 period, while Manurewa showed a decline in the number of unsocialised adult stray cats euthanased over the three study years, in contrast to the juvenile cats, for which there was a decrease in 2015/2016 and then a slight increase in 2016/2017. In other areas, Mt Roskill in Central Auckland and Henderson in West Auckland were suburbs of origin for more adult and juvenile unsocialised stray cats euthanased compared to the surrounding areas (Figure 4).

### 3.5. Comparison between Suburbs

The percentage change in the number of incoming stray adult and juvenile felines, neonatal/underage euthanasias, unsocialised stray cats euthanased, and unsocialised stray cats sterilised and returned were all compared between Manurewa and the average for these outcome measures for the non-targeted suburbs as one group (Table 1). Overall, there was a reduction in all of these parameters. There was a reduction in incoming adult felines from Manurewa of 39%, while the average for the non-targeted suburb group was a 17% increase in incoming adults. There was a reduction in incoming juvenile felines from Manurewa of 17%, while the average for the non-targeted suburb group was a 43% increase. There was a reduction in neonatal/underage euthanasias from Manurewa of 34%, while the average for the non-targeted suburb group was a 43% increase. There was a reduction in unsocialised stray cats sterilised and returned (separately to the TTNR programme) from Manurewa of 7%, while the average for the non-targeted suburb group was a 100% increase. There was a reduction in unsocialised stray cats from Manurewa euthanased (separately to the TTNR programme) of 47%; the average for the non-targeted suburb group was a 13% increase. The percentage change reduction in all of the outcome measures for Manurewa was found to be significantly greater (*p* < 0.01) compared to the non-targeted suburb group.

#### 3.5.1. Kernel Density Surface Models

The kernel density maps indicate areas where high values cluster and where the surrounding areas have higher numbers compared to others. The kernel density maps indicate the density of features or numbers of counted variables (such as cat intake) in a neighbourhood. As indicated in the legend, higher density areas show up in red, whereas low density areas are blue, with shading through yellow for intermediate densities.

**Figure 5:**
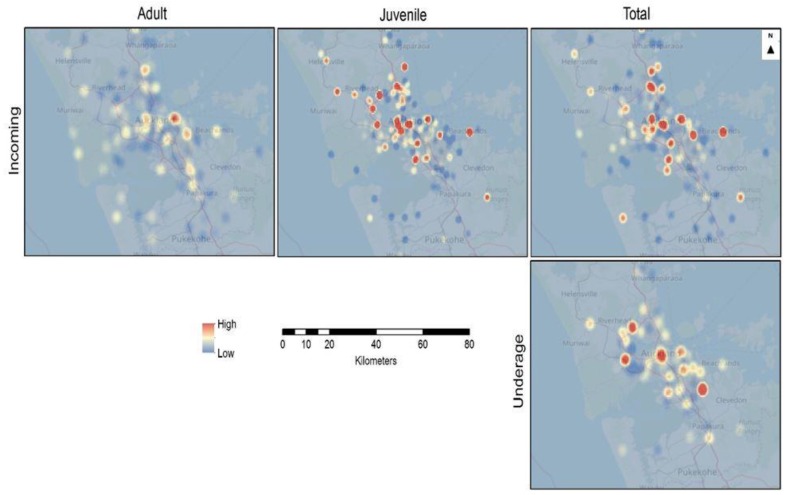
Kernel density map of Auckland showing the percentage change for the origin of incoming adults, juveniles, and total stray cats, and neonatal/underage euthanasias during the 2014/2015, 2015/2016, and 2016/2017 financial year periods.

Kernel density surface models of the percentage change in the numbers of incoming adults, juveniles, and total adults and juveniles, as well as neonatal/underage euthanasias, show that juvenile cats have higher percentages changes in more areas compared to the adults and neonatal/underage euthanasias. Most of the juvenile high-density percentage sites are located in Central Auckland. For the neonatal/underage euthanasias data, sites with high percentage changes are observable at the Central and Western areas of the city (Figure 5).

**Figure 6:**
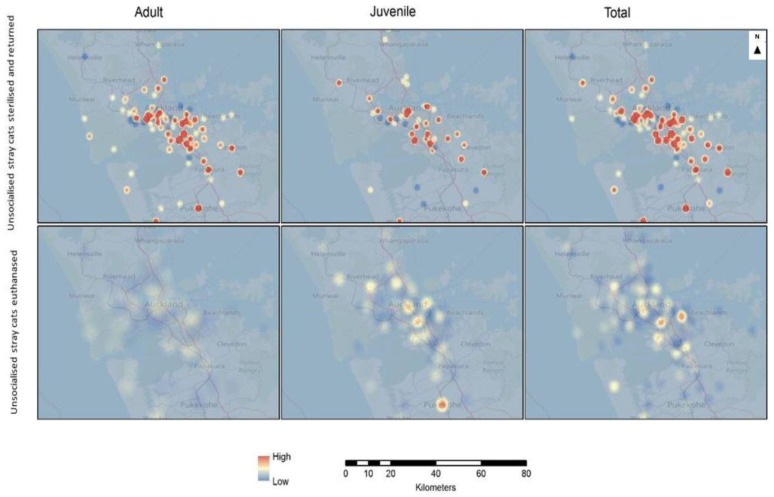
Kernel density map of Auckland showing the percentage change for the origin of unsocialised stray cats euthanased and unsocialised stray cats sterilised and returned through the shelter during the 2014/2015, 2015/2016, and 2016/2017 financial year periods.

The kernel density surface model of percentage change for unsocialised stray cats sterilised and returned shows that the number of adults sterilised and returned has increased in more areas than for juveniles, with greater changes mainly in the Central Auckland area. The unsocialised stray cats euthanased percentage change is much less in terms of magnitude when compared to the unsocialised stray cats sterilised and returned in adults, juveniles, and total percentage change surface densities (Figure 6).

#### 3.5.2. Hotspot Analyses

The hot spot maps indicate statistically significant clustering using a geostatistical measure (Getis-Ord Gi* statistic) that characterises the counts for each of the outcome measures in terms of the values of neighbouring data points [57], and identifies points with high values surrounded by points with similarly high values; these are designated as hot spots (in red). Conversely, low values surrounded by low values are designated as cold spots (in blue). The statistical significance of each point in terms of its neighbourhood values results provides the basis for determining a level of confidence that a particular location is a hot spot, a cold spot, or that there is no significance in terms of this measure (as indicated in the legend).

**Figure 7:**
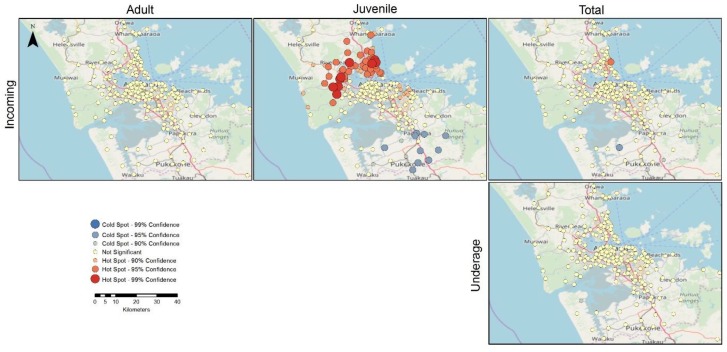
Hotspot and cold spot maps of Auckland showing percentage change for the origin of incoming adults, juveniles, and total stray cats, and neonatal/underage euthanasias during the 2014/2015, 2015/2016, and 2016/2017 financial year periods.

Results of hotspot analysis of percentage change for the origin of incoming cats show that Western Auckland and the North Shore areas have neighbourhoods that are contributing significantly similar high numbers of incoming juveniles. Conversely, identified cold spots in South Auckland indicate that low values of percentage change are consistent or near each other within that neighbourhood. For adult cats, there are no significant hot or cold spots identified. When the total count is analysed, there is only one hot spot near Brown’s Bay in the North Shore and a cold spot at Kingseat in South Auckland going towards Clark’s Beach (Figure 7).

**Figure 8:**
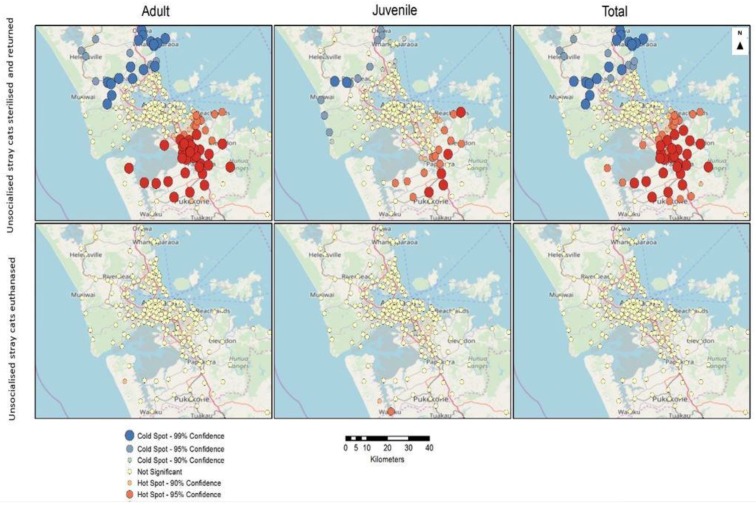
Hotspot and cold spot maps of Auckland showing percentage change for the origin of unsocialised cats sterilised and returned and unsocialised cats euthanased during the 2014/2015, 2015/2016, and 2016/2017 financial year periods.

Results of hotspot analysis of percentage change for the origin of unsocialised cats sterilised and returned showed clustering of hotspots and cold spots in the areas of North Auckland and South Auckland, respectively. The percentage change for the origin of unsocialised cats euthanased did not show significant hotspots or cold spots, except for Waiku, where one hotspot for juveniles is found (Figure 8).

## 4. Discussion

The number of incoming stray felines, underage euthanasias, and unsocialised adult stray cat euthanasias all reduced considerably for the suburb targeted by the TTNR pilot (Manurewa) after the programme. These changes were significantly greater than the corresponding averages for the non-targeted suburb group. Although TTNR has the potential to result in positive change in all of these outcome measures and the results from this preliminary study are encouraging, the results must be interpreted with caution due to the short term nature of this pilot programme and the coexistence of other factors that may have contributed to, or account for, the changes in outcome measures. These factors include: normal variation, climatic variation and its effect on cat reproduction, concurrent free sterilisation programmes conducted in various suburbs throughout Auckland, and activities of other welfare organisations and cat rescue groups. Due to the complex nature of communities and the many factors that influence shelter statistics and cat populations, it is very difficult to definitively demonstrate or assess if there is a causal relationship between changes in shelter statistics or cat populations and TTNR programmes (or any cat management strategy). Reductions in shelter cat intake and euthanasias associated with implementation of TNR have been documented by other researchers [5,48]. Other studies have documented decreases in cat numbers and colony size associated with TNR programmes, rather than shelter parameters [46,47,59]. The results of the current study suggest that TTNR could potentially be an effective tool in a multifaceted holistic approach to reduce incoming stray felines, underage euthanasias, and unsocialised stray cat euthanasias in Auckland. However, the shelter also ran free sterilisation programmes for owned cats during the same time as the TTNR pilot. These programmes were targeted to areas of Auckland from which the shelter was receiving the largest number of cats. It is not possible to know what contribution these had on the shelter outcomes measured, but it is likely that at least some of the positive change in measured parameters can be attributed to the free sterilisation programmes (and other background activities from other organisations that may also have impacted on stray cat numbers), in addition to the TTNR pilot. This means that the improvement in measured parameters cannot be definitively attributed to the TTNR programme, and so the results, although encouraging, need to be interpreted with this in mind. In future research, data on other background sterilisation programmes and activities that might be likely to impact on stray cat numbers should be assessed as potential confounding factors when assessing TNR programmes.

A number of simulation models and studies have produced estimates of the number of cats that would need to be sterilised to effectively control a cat population. McCarthy et al.’s [8] model estimated that over 82% of cats in a population of 200 cats would need to be removed to result in elimination of the population over 4000 days; Nutter [47] estimated that sterilisation levels of at least 75% to 80% would be necessary to cause population decline and eventual colony extinction, assuming that immigrant cats are also sterilised; Andersen et al. [60] estimated that annual sterilisation of >75% of the fertile population was required. Budke and Slater’s model [39] estimated that cessation of cat population growth would require surgical sterilisation of more than 51% of adult and juvenile (<1 year) intact female cats annually, and having approximately 71% of the total female population sterilised at all times. Although the cat population of Manurewa was unknown at the time of the study, and so a calculation of the proportion of the cat population sterilised by the programme was not possible, it must be noted that this was a pilot study, and the number of cats sterilised as a proportion of the population was likely to be significantly lower than the target proportions listed above. This assessment is based on a comparison of the number of cats sterilised per 1000 human residents in the suburb to Levy at al.’s [5] recent and larger study that demonstrated a rapid reduction in shelter intake and euthanasia related to a TNR programme, in which 54% of the projected community cat population in the targeted area were captured and sterilised as part of the TNR program over the 2-year study period [5]. In the current study, 4.2 cats/1000 residents were sterilised as part of the pilot programme, substantially less than the 60 cats per 1000 residents sterilised in Levy et al.’s 2014 study (the large different in numbers of cats/1000 residents could be explained by the pilot nature of this programme, funding differences, and the requirement that cats would only be sterilised and returned with the consent and participation of the resident where the cat resided). There was a lower cat intake of cats from Manurewa into the participating shelter in the current study compared to the intake reported in the Levy study (5 incoming cats/1000 residents compared to 14 cats/1000 residents in the Levy et al. study [5]). It is not unreasonable to assume that a larger scale programme, which sterilised more cats per 1000 residents and a larger proportion of the cats in the area, would have a greater impact than that seen in this study. In future programmes building on this preliminary pilot study, it is suggested that a sterilisation rate of at least 60 cats per 1000 residents is targeted, since this resulted in significant decreases in intake and euthanasia for the targeted suburb compared to the non-targeted suburb in the Levy study [5]. However, further research is needed to determine if intake per 1000 residents can be used as an indicator of the sterilisation rate required to produce a significant decrease in shelter intake and euthanasia. If comparing TTNR and lethal cat control programmes, it is important to consider that a lethal control programme would need to kill a high proportion of the cat population and be maintained over a long period of time, just as in a TTNR programme a high proportion of cats would need to be sterilised and this maintained over a long period of time [9,47]. Trap and kill programmes are far from simple to implement effectively and involve significant investments of resources to have any chance of success; this has been demonstrated from programmes to eradicate cats even from geographically isolated islands, which have fewer complications compared to programmes in urban and non- geographically isolated areas (for example, cat immigration and anthropogenic interactions) [61]. Furthermore, there is evidence that less than optimum cat removal rates may actually lead to an increase in cat numbers [62]. The socio-political and practical implications of cat management programmes must also be taken into account when considering which are the most viable options, particularly in urban areas in which the community may not be supportive of lethal programmes, as community support and adequate resourcing are key to success [3,44]. The increase in unsocialised juvenile stray cat euthanasias in Manurewa after the TTNR programme was unexpected, and the reason for this is unknown. One explanation could be related to the short time frame of the study; it would be expected that, over a sufficient amount of time, neonatal and juvenile cat intake and euthanasia would be the most impacted by TTNR programmes, as there would be fewer kittens born over time and, consequently, a decline in intake and euthanasia. It is likely that the one year TTNR programme and assessment was not long enough to result in those expected changes, and that the increase in unsocialised juvenile stray cat euthanasias in Manurewa was just a result of normal variation. This highlights the need for TTNR programmes to be long term consistent programmes, with adequate resourcing as has been recommended [37,45,63], rather than short term and ad-hoc. In addition, if a greater proportion of the stray cat population had been sterilised this would likely have resulted in a more marked and rapid effect on juvenile stray cat euthanasias and other parameters.

Including adoption is considered to be one of the elements of a successful TNR programme [5,11,37,46]. The TTNR pilot reported here included an adoption component for suitable cats; 58 juvenile cats were rehomed (0.7/1000 residents) as part of the pilot, and the adoption component was likely a contributing factor in the programme’s success. It is advisable that, where possible, an adoption component for suitable cats always be included in a TTNR programme.

The data highlighted that South Auckland in particular represents a significant source of stray cats in the Auckland region. This is consistent with previous research in Auckland showing significant clustering of stray cats in South Auckland compared to elsewhere in the region [54]. Other research has shown a positive correlation between stray cat density and socioeconomic deprivation [7,54,64], highlighting the need to address other societal issues that are likely interrelated. Using spatial mapping techniques, such as those used in this study, is useful for identifying areas that need programmes to address issues such as large numbers of stray cats in a community [54,55] and can assist in targeting resources to these specific geographical areas where they are most needed. Spatial mapping techniques may also be helpful when seeking funding for programmes to manage cat populations, as they provide concise and easy to understand visualisation of the relevant data that can be presented to potential stakeholders and funders. However, not all areas in a community will have people bringing cats to the shelter, and these cats will, consequently, not be captured by shelter data, or spatial mapping using shelter data. It is also probable that some of those areas that will not appear in shelter data, because people do not bring cats from those areas into the shelter, are likely the areas that are least served and most in need of assistance to manage their cat populations. This highlights that, if the goal is decreasing the number of stray cats in the environment, shelter data may not provide all the information needed to make cat management decisions. On the ground work to identify locations with the most cats or locations that most need assistance or that would most benefit from TTNR programme will be important in determining accurately where the areas of concern and high stray cat populations are in a community. These issues should be considered in future research and when considering how to decide on cat management strategies and allocation of resources to these within a community.

In this TTNR pilot programme, 1.9% of cats were euthanased due to health problems. This is a euthanasia rate that sits between the 0.4–0.5% reported for two TNR studies in the US [5,65], and the 4% [63] and estimated 5–10% [37] euthanasia rates for two other TNR programmes, again both in the USA. There were no peri-surgical deaths during the Auckland TTNR pilot programme, while other programmes have reported a peri-surgical death rate of 0.3–0.4% [5,65,66], which is comparable to that reported for companion cat sterilisation surgeries [67] and high volume spay-neuter clinics [68]. In this study, there was one cat that was recaptured and treated for a post-operative wound complication; this equates to a wound complication rate of 0.29%. This is lower than other reported wound complication rates in shelter cats undergoing ovariohysterectomy (6.09% [69]) and elective surgery (including ovariohysterectomy) in the general companion animal population (2.2–5.7% [70,71]). However, the number of peri-operative deaths and complications will presumably increase with increasing numbers of cats operated on; since the other reported programmes sterilised larger numbers of cats compared to the Auckland pilot programme, it is likely that this explains the difference in incidence of peri-operative mortality and morbidity. Although the evidence from this study and others indicates low peri-operative morbidity and mortality, it is not possible to know with certainty that no cats died or that other cats did not have peri-operative complications without retrapping all the stray cats sterilised. Retrapping all the cats to check for complications is not ethically acceptable given the additional stress and the potential for cats to be injured during this process. Therefore, the potential that the peri-operative morbidity and mortality is higher than reported has to be balanced against the benefit to the population as a whole. However, where possible it would be of benefit to record and report on peri-operative morbidity and mortality in situations where this can be done without causing stress or harm to the cats (for example, in intensively managed colonies where carers would be able to consistently monitor the sterilised and released cats).

This TTNR pilot programme was assessed using the number of incoming stray felines, neonatal/underage euthanasias, unsocialised stray cats sterilised and returned, and unsocialised stray cat euthanasias at the main animal shelter receiving cats in Auckland. There are other parameters that could be considered as measures of success that were not documented during this study. These include measures of the impact of cats on wildlife, cat welfare measures, and cat numbers in the targeted suburb before and after the programme [3,46,59,72]. However, since this pilot study did show positive outcomes in terms of the parameters measured, it would be worth expanding the programme and measuring additional parameters such as those suggested here. This pilot study evaluated a TTNR programme over a relatively short period of time (one year), and some of the beneficial outcomes of such a programme might only be expected to come to full fruition after some years (for example, a reduction in stray kitten numbers and stray cat-related nuisance problems). However, the time frame for seeing beneficial outcomes is likely also related to the proportion of cats sterilised in an area; if a higher number of cats are sterilised, it is likely that there will be a more rapid reduction in intake from the target area and in nuisance calls, as demonstrated in other research with higher sterilisation rates [5]. It is valuable to critically evaluate TTNR programmes prospectively over the medium to long term to fully appreciate their impact. In addition to utilising other measures of programme outcomes in future research, it is important to consider that shelter-based measures may be subject to modification from factors that may be related to cat management programmes, rather than being due to reduced stray cat numbers as a direct result of these programmes. For example, the numbers of stray cats brought to the shelter by members of the public may decrease as a result of community awareness of the TTNR programme, leading to people deciding not to bring stray cats into the shelter. In this scenario, the shelter intake numbers for stray cats may decrease, but there may not actually be a decrease in stray cat numbers (at least in the short term). Therefore, these issues should be considered in future research. Another limitation of this pilot study was that the numbers from the targeted suburb had to be compared to an average of those from all the other non-targeted suburbs, because the sample size was not large enough to compare each of the non-targeted suburbs to Manurewa individually. In addition, the location of origin of the cats was only recorded as the suburb rather than the exact address; in future research, more detailed locations could provide more accurate information to inform shelter policy and TTNR assessments. Auckland is a large and diverse city, and so the approach taken, although necessary for this pilot study, could be improved in future research; with larger numbers of cats, the parameters could be compared between each of the suburbs with and without TTNR programmes.

Significant cost was involved in implementing the TTNR programme, as has been reported by other researchers [9,46]. A cost benefit analysis in which the cost of housing and rehoming or euthanasing similar numbers of cats as previously compared to the reduced numbers after the TTNR programme would provide useful information to inform financial resourcing of cat management options. However, some benefits of a strategy such as a TTNR programme cannot be financially measured; there can be benefits in terms of the welfare of the sterilised cats [47,73,74]; community satisfaction as a result of both reduced community nuisance and use of a more publically acceptable method of managing cat numbers [44,75]; reduced costs associated with animal welfare; and shelter staff and volunteers having to be involved with lethal cat control, as these people may suffer from moral stress as a result, and this can lead to health problems and high staff/volunteer turnover [22,23,25]. Although a high percentage of cats in an area need to be sterilised, and effective mitigation of immigration of new cats into the area must be achieved for a TTNR programme to be successful [45,46,63,76], it must be highlighted that a lethal cat management programme would also need to consistently achieve high removal rates for a prolonged period and effective mitigation of immigration of new cats into the area to have the potential to markedly reduce cat populations [8,10,38,39]. A lethal programme would also require significant investment of resources if it were to achieve the removal rates that computer modelling indicates would be necessary [8,10,38]. To the authors’ knowledge, there have been no studies that document a successful intensive and targeted trap and kill programme in an urban area, and which compare the costs of such a programme to a similar non-lethal programme such as a TTNR programme.

This pilot programme demonstrates that TTNR has the potential to contribute to a reduction in numbers of incoming stray felines, underage euthanasias, and unsocialised stray cat euthanasias in an urban New Zealand setting. However, TTNR is a resource-intensive cat management tool, and to make the investment worthwhile it is important that a specific and appropriate area is targeted, and a 70–80% sterilisation rate must be achieved in the target area to result in a decrease in the cat population [8,9,10]. The programme must be implemented well, repetitively, and consistently [77]. In addition, cat immigration/abandonment must be monitored, and any new cats must be sterilised or adopted before they can reproduce [30,77,78]. Such a programme needs dedicated resources and staffing and is a long-term strategy that should reduce cat numbers and impact over time [3,77]. A TTNR programme that is implemented poorly in an inappropriate area is likely to be a waste of resources and not achieve the desired objectives. However, even deciding on the desired objectives can be a complex and controversial task [45], as these are likely to vary for different stakeholders [3,79]. For example, the objectives for animal welfare organisations and cat advocates may include some or all of the following: improved cat health and welfare, a stable or reducing cat population, and fewer cat admissions and euthanasias in animal shelters [79,80]. In contrast, the objectives for conservationists and conservation biologists may be complete and rapid extinction of a cat colony and reduction or elimination of cat predation on wildlife [79,81,82]. Other stakeholder groups are likely to have yet other objectives and priorities, potentially including reduction in nuisance from stray cats, reduction in spending on cat management, and less public concern and outcry about cat issues [3]. TTNR should be considered as one cat management tool that can be of benefit in conjunction with other appropriate strategies, but the choice of cat management tools needs to be assessed on a case by case basis for the individual area and situation. Such an assessment could utilise a framework such as that proposed in the international consensus principles for ethical wildlife control. These principles recommend that any efforts to control wildlife ‘should begin wherever possible by altering the human practices that cause human—wildlife conflict and by developing a culture of coexistence; be justified by evidence that significant harms are being caused to people, property, livelihoods, ecosystems, and/or other animals; have measurable outcome-based objectives that are clear, achievable, monitored, and adaptive; predictably minimise animal welfare harms to the fewest number of animals; be informed by community values as well as scientific, technical, and practical information; be integrated into plans for systematic long-term management; and be based on the specifics of the situation rather than negative labels (pest, overabundant) applied to the target species’ [83].

Another option for humanely managing cat populations in urban areas is managed TTNR, in which unowned cats are provided with systematic and ongoing care, as well as being sterilised. This approach is similar to that reported in the Newburyport, Massachusetts TNR case study [37]. Intensive public and stakeholder engagement, feeding, and monitoring/caring for cats was part of the Newburyport programme, in addition to TNR being used to systematically and comprehensively sterilise all of the stray cats in the targeted area. The general health of the cats in the Newburyport programme improved over time, which is consistent with other reports that cat health improves with sterilisation and increased care [31,47,73,74]. Furthermore, there were no longer any unowned cats residing in the targeted area after 17 years of the Newburyport programme. Managed TTNR has been suggested as a humane cat management tool for New Zealand [3], and it would seem to be an option that should be explored further, given the potential benefits to cat welfare, as well as the potential to reduce cat numbers and impact.

## 5. Conclusions

This pilot programme suggests that TTNR could be a useful and positive cat management tool in urban New Zealand. Further assessment of such programmes with larger numbers of cats, as well as sterilisation of a greater proportion of the stray cat population, incorporating other outcome measures and potential confounding factors over a longer time period, would help inform the cat management discussion in New Zealand and internationally. 

## Figures and Tables

**Table 1:** Percentage change in the number of incoming stray adult and juvenile felines, neonatal/underage euthanasias, unsocialised stray cats euthanased, and unsocialised stray cats sterilised through the shelter that originated from Manurewa (the suburb targeted by the TTNR programme), and the average for these outcome measures for the non-targeted suburb group.

Outcome Measure from the Shelter	Manurewa (the Targeted Suburb)	Average for Non-Targeted Suburb Group
	Percentage change	
Incoming adult felines	−39 *	+17
Incoming juvenile felines	−17 *	+43
Neonatal/underage euthanasias	−34 *	+43
Unsocialised stray cats sterilised and returned (adults and juveniles combined)	−7 *	+100
Unsocialised stray cats euthanased (adults and juveniles combined)	−47 *	+13

* Indicates that the percentage change reduction in Manurewa was found to be significantly greater (*p* < 0.01) than for the average for the non-targeted suburb group.
